# Casimir Effect in MEMS: Materials, Geometries, and Metrologies—A Review

**DOI:** 10.3390/ma17143393

**Published:** 2024-07-09

**Authors:** Basma Elsaka, Xiaohui Yang, Philipp Kästner, Kristina Dingel, Bernhard Sick, Peter Lehmann, Stefan Yoshi Buhmann, Hartmut Hillmer

**Affiliations:** 1 Institute of Nanostructure Technologies and Analytics (INA), Technological Electronics Department, University of Kassel, Heinrich-Plett-Straße 40, 34132 Kassel, Germany; elsaka@ina.uni-kassel.de (B.E.); yang@ina.uni-kassel.de (X.Y.); kaestner@ina.uni-kassel.de (P.K.); 2 Institute for Systems Analytics and Control (ISAC), Intelligent Embedded Systems Department, University of Kassel, Wilhelmshöher Allee 71-73, 34121 Kassel, Germany; kristina.dingel@uni-kassel.de (K.D.); bsick@uni-kassel.de (B.S.); 3 Artificial Intelligence Methods for Experiment Design (AIM-ED), Joint Lab between Helmholtz-Zentrum für Materialien und Energie, Berlin (HZB) and the University of Kassel, 34121 Kassel, Germany; 4 Measurement Technology Group, Faculty of Electrical Engineering and Computer Science, University of Kassel, Wilhelmshöher Allee 71, 34121 Kassel, Germany; p.lehmann@uni-kassel.de; 5 Center for Interdisciplinary Nanostructure Science and Technology (CINSaT), Heinrich-Plett-Straße 40, 34132 Kassel, Germany; 6 Institut für Physik, University of Kassel, Heinrich-Plett-Straße 40, 34132 Kassel, Germany; stefan.buhmann@uni-kassel.de

**Keywords:** dispersion forces, Casimir effect, Van-der-Waals force, retardation, surface roughness, self-assembly, attraction and repulsion, nanocavities, solid–fluid interfaces

## Abstract

Casimir force densities, i.e., force per area, become very large if two solid material surfaces come closer together to each other than 10 nm. In most cases, the forces are attractive. In some cases, they can be repulsive depending on the solid materials and the fluid medium in between. This review provides an overview of experimental and theoretical studies that have been performed and focuses on four main aspects: (i) the combinations of different materials, (ii) the considered geometries, (iii) the applied experimental measurement methodologies and (iv) a novel self-assembly methodology based on Casimir forces. Briefly reviewed is also the influence of additional parameters such as temperature, conductivity, and surface roughness. The Casimir effect opens many application possibilities in microelectromechanical systems (MEMS) and nanoelectromechanical systems (NEMS), where an overview is also provided. The knowledge generation in this fascinating field requires interdisciplinary approaches to generate synergetic effects between technological fabrication metrology, theoretical simulations, the establishment of adequate models, artificial intelligence, and machine learning. Finally, multiple applications are addressed as a research roadmap.

## 1. Introduction

In 1948, the Dutch physicist Hendrik Casimir came up with a fascinating idea that in a vacuum, attractive forces would appear between two neutral (uncharged), ideally conducting and reflecting metal plates [1]. Using a simple model but complex physics, including the best of modern physics of the last century (see below), he derived an amazingly simple analytical formula in which the Casimir force, F_c_, depends only on the area of the plates *A* and their separation distance *d*: F_c_ = π h c/480 A/d^4^ [1], where h and c denote the Planck constant and light velocity, respectively

**Figure 1:**
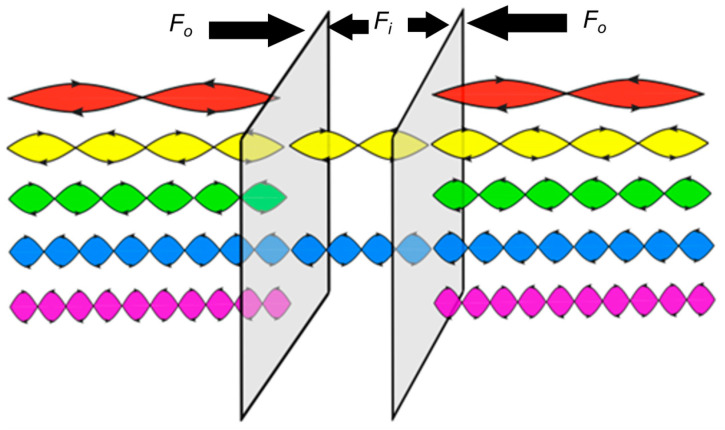
Schematic illustration of Casimir forces between parallel plates using the quantum electromagnetic model. F_0_ represents the forces exerted on the plates due to the quantum waves outside the plates, and F_i_ refers to the one in between the plates.

The idea and the derivations were complex and not as simple as this formula. Casimir used the zero-point energies of all oscillators (non-vanishing zero-point energy), quantum theory, Heisenberg’s uncertainty relation and the wave–particle duality. Casimir explained this force by comparing the zero-point pressure of electromagnetic waves inside and outside the cavity. Using the wave–particle duality energies and assuming a smaller photon density of states inside the cavity than outside, then less momentum transfer to the plates occurs from inside compared to outside. This results in net pressure (forces per area) from outside of the plates, as shown in Figure 1. An intuitive explanation is repeated here: the number of allowed optical modes inside is far smaller than outside (difference in the optical density of states). Using Einstein’s mass–energy equivalent and quantum fluctuations of the electromagnetic fields results in a virtual particle pressure on two metal plates. However, due to the difference in the optical density of states, the force inside *F*_i_ is smaller than the force outside *F*_o_. The pressure (force per area) from outside is higher than from inside, generating an attraction between the two plates.

The formula is an extension of the calculation of interaction between a perfectly conducting plate and an atom or a molecule, which was presented by Hendrik Casimir and Dirk Polder in their study, based on the influence of retardation on the (London)–(Van der Waals) force [2]. The retardation means that at an appreciable distance, the time for an instantaneous dipole to interact with a neighbouring atom is comparable to the lifetime of the dipole. The interaction is potentially out of phase, reducing the attractive force. Thus, the Casimir force was thought to be closely related to the retarded Van der Waals attraction initially at long separations (several nanometres to several micrometres). In contrast, the Van der Waals force is non-retarded at close separations (several angstroms to nanometres). Between approximately 5 nm and 100 nm separation, there is a transition region from Van der Waals force to Casimir force [3,4,5]. Van der Waals and Casimir forces are typically attractive within the scale in which they exist, while Casimir forces sometimes exhibit a repulsive nature and are strongly dependent on the geometrical shapes, surface roughness, permittivity and conductivity of the materials, temperature and other conditions [6,7].

Up to now, many theoretical model calculations and experiments with different geometries have been studied. In 1956, Lifshitz developed a theory of molecular attractive forces between any shaped solids at any temperature, and then, Błocki et al. concluded that the Proximity Force Approximations (PFA) treating small distances, which showed the force between two gently curved objects with the same material, is proportional to the interaction potential per unit area between two flat surfaces [8,9]. Their theoretical calculations have made a significant contribution to the study of Casimir forces. In the following sections, we describe the Casimir force from four aspects: different interacting geometries, main measurement methods, corrective calculations with critical parameters, and influences on the characteristics and applications of MEMS/NEMS devices.

There are plenty of books [6,7,10,11,12,13,14,15], book chapters [16] and extended papers [17,18,19,20,21,22,23,24] that provide a good overview of the whole field. They have the characters of textbooks, tutorials, reviews or surveys, or a combination of them. All of them are more or less different from each other since they have their own focus, and each considers the progress of research in a specific time period.

In the following, an overview of a selection of reviews is given, mentioning their specific foci. One key challenge in this field has been the lack of complete agreement between theoretical model calculations and corresponding experimental data. Experimental groups have been striving to improve systematic and statistical errors, while theoretical groups have focused on refining their models by incorporating corrections for non-idealities and higher-order effects. In 2004, a review was published of effects relevant to measurements of the Casimir force between real materials, discussing the Casimir force between parallel plates that have been rederived using a strong coupling limit of δ-function potential planes [17]. In 2005, the theory of Casimir forces focused on the corrections for real materials and finite temperatures [18].

Further on, in 2009, a review dealt with the continuing controversy on how to incorporate thermal corrections into the Casimir force between real metallic plates [19,21]. It also addresses the aspect of real materials and temperature influence and is devoted to the 60-year Casimir effect. There was a review in 2011 describing specific MEMS sensors to detect the Casimir interaction. The paper summarises the most recent experimental results. In addition, it suggests potential optomechanical experiments allowing the measure of this force in regimes that are currently unreachable [16]. Another survey in 2011 focused on the decades-long search for geometries, which revealed Casimir forces, which are non-attractive and non-monotonic [20]. A new understanding of complex microstructured geometries has been obtained. In addition, stable suspension of objects with unusual non-additives is investigated. Temperature effects are also studied here. Repulsion effects between objects in fluids were found, as well as non-additive forces in nano-trenched surfaces. The review also refers to the influence of new material choices.

In later years, more studies followed up. Starting in 2016, a survey on quantum friction due to lateral Casimir forces was published. This paper deals with dissipative quantum electrodynamics [21]. To the best of our knowledge, no experiments are existing up to now. In 2020, a review appeared focussing on the 50-year dynamic (or non-adiabatic) Casimir effect [22]. In 2021, a review discussed certain developments in Casimir physics in novel 2D materials, Chern and topological insulators [23]. Also in 2021, the last 10 years on Casimir interaction in electronic topological materials focusing on solids having surface or bulk electronic band structures with nontrivial topologies was covered. Three-dimensional magnetic topological insulators, two-dimensional Chern insulators and graphene monolayers exhibiting the relativistic quantum Hall effect and time reversal symmetry-broken Weyl semimetals have been considered [24].

There are also a large number of reviews focussing on the comparison of the results of experimental studies and theoretical model calculations by Mostepaneko et al. [15,25,26,27,28]. In most of the cases, theoretical groups were performing line shape fits to experimental data (force-distance measurements) reported in the literature. For example, semiconductor test bodies are used to measure the Casimir force in a setup including an Au-coated sphere and different semiconductor surfaces. Thus, one metallic plate is replaced by a semiconductor plate, causing significant changes in the forces. The doping levels, i.e., the concentration of charge carriers in the semiconductor material also play an important role. Furthermore, the Casimir force changes between an Au-coated sphere and a Si plate with and without laser light illumination had been measured. In addition, the Casimir force gradient was measured between an Au-coated sphere and a Si plate with different rectangular corrugations (different grating types). For different experiments and analyses, the review also compares methodologies and discusses advantages and disadvantages. These are valuable tests if the Lifshitz theory of Van der Waals and Casimir forces, supplemented by several corrections, matches these materials. Further studies were reported, where experiments and simulations have been performed by the same group, as well as the comparison between them [29,30].

This review focuses on four main aspects: (i) the combinations of different materials, (ii) the considered geometries, (iii) the applied experimental measurement methodologies and (iv) a novel self-assembly methodology based on Casimir forces. This paper provides a survey of our research concerning the application of Casimir forces for self-assembly.

## 2. Survey about Experimental and Theoretical Studies of Casimir Forces

**Figure 2:**
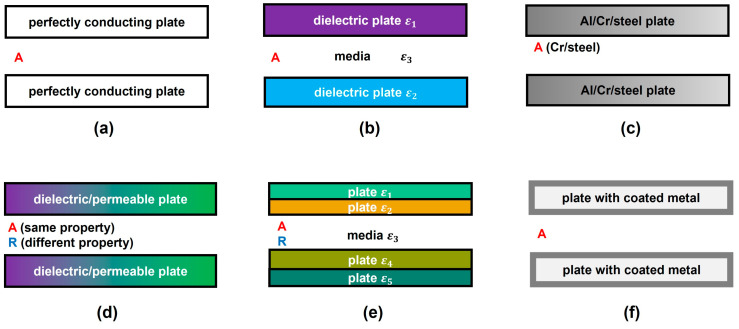
Studies of Casimir force on different parallel plates. (**a**) Perfectly conducting plates [1], (**b**) dielectric plates [8,32], (**c**) metal plates [33], (**d**) dielectric and infinitely permeable plates [34,35,36], (**e**) multi-layered plates [37,38,39,40], and (**f**) metallic plates [41,42].

**Figure 3:**
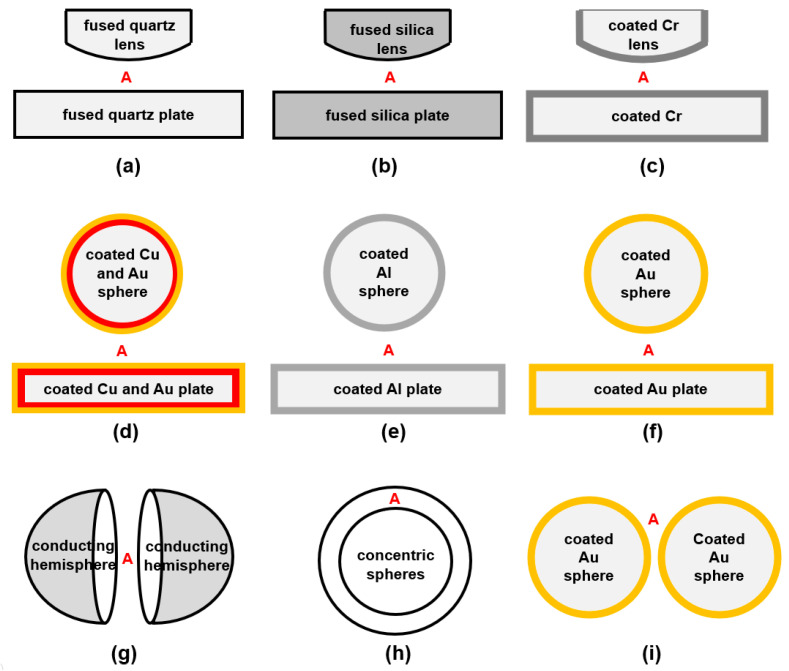
Studies of Casimir forces on spheres/lens–plates and sphere–sphere geometries. (**a**) Quartz lens–plate [43,44], (**b**) silica lens–plate [45,46], (**c**) coated Cr lens–plate [47], (**d**) coated Cu and Au sphere–plate [48], (**e**) coated Al sphere–plate [49,50], (**f**) coated Au sphere–plate [51,52,53], (**g**) conducting hemispheres [57], (**h**) goad-coated spheres [59], (**i**) concentric spheres [58].

**Figure 4:**
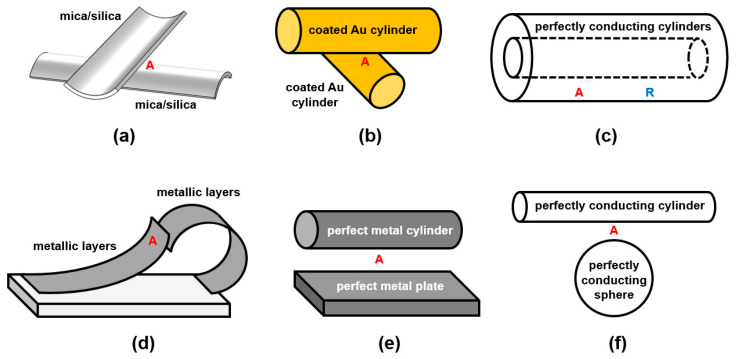
Studies of Casimir forces on cylinders with different geometries. (**a**) Crossed cylinders of mica and silica [3,45,60], (**b**) crossed cylinders of gold [61], (**c**) perfectly conducting, parallel cylinders [62,63], (**d**) cylindrically bent metallic blades [64,65,66], (**e**) cylinder–plate of perfect metals [67,68], (**f**) perfectly conducting cylinder–sphere [69] (completely redrawn by the ideas of these references).

**Figure 5:**
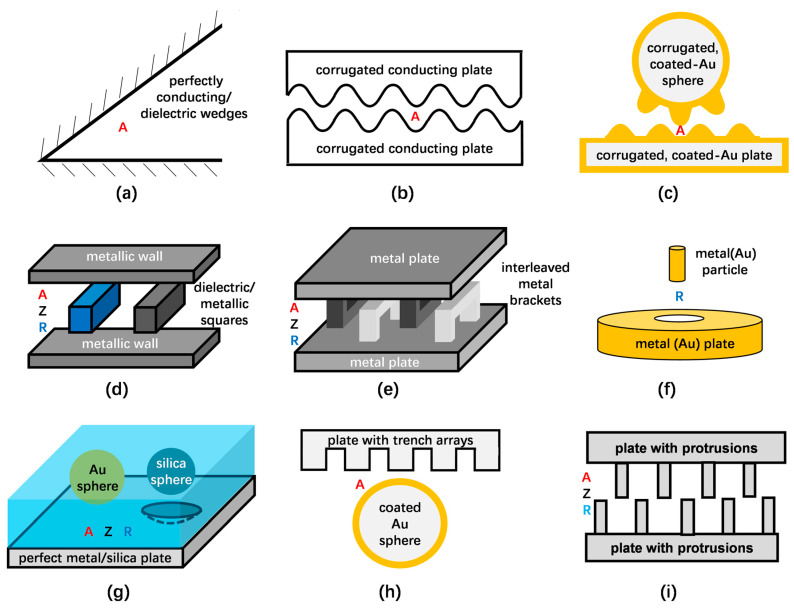
Different geometries of theoretical studies about Casimir forces. (**a**) Perfectly conducting and dielectric wedges [70,71,72], (**b**) corrugated plates [73,74], (**c**) corrugated sphere–plate [75,76], (**d**) squares between two walls [77], (**e**) parallel metal plates with interleaved brackets [78], (**f**) metal particles above plate with a hole [79], (**g**) sphere–plate immersed in liquid [55,56,80,81], (**h**) silicon plate with trench arrays and gold sphere [82,83], (**i**) parallel plates with protrusions [84,85] (completely redrawn by the ideas of these references). The following abbreviations are used: attractive force (A), repulsive force (R) and a force of zero (Z).

Unique to the Casimir force is its strong dependence on geometrical shapes [20,31]. Depending on various geometries, this review divides these studies into several geometry groups: (1) plate–plate, (2) lens/sphere–plate and sphere–sphere, (3) cylinder–cylinder/plate/sphere, and (4) special geometries. Sketches of the groups are shown in Figures 2–5. The letters A, R and Z denote the attraction, repulsion and the case of zero interaction.

### 2.1. Plate–Plate Geometries with Different Material Combinations

Casimir first proposed the prediction of attraction force between two perfectly conducting plates, as Figure 2a shows [1]. After that, Lifshitz obtained the general representation of the attractive Van der Waals and Casimir force in terms of the frequency-dependent dielectric permittivity *ε* of different media [8], which was confirmed by Schwinger et al. [32], as shown in Figure 2b. Next, Spanaary observed attraction between chromium and steel plates but repulsion between two aluminium plates in his measurements, as depicted in Figure 2c. He predicted that inaccurate results were caused by the thin sheets of the oxide layer [33]. Then, Figure 2d illustrates studies from Boyer on the Casimir effect between two dielectric and permeable plates. He noticed that the force was attractive and repulsive when the main properties of plates were the same and different, respectively [34]. Kupiszewska and Mostowski also mentioned that the interaction force was attractive when the signs of reflection coefficients were the same while repulsive when they were opposite [35]. Recently, this conclusion was proven by Høye again [36]. Figure 2e shows multilayered plates with different permittivity *ε*, and the Casimir force was shown to be attractive or repulsive by configuring diverse layer structures and materials [37,38,39,40]. After that, Figure 2f shows two silicon surfaces with coated metal (chromium or gold). Attractive forces between them were measured, which was in good agreement with Casimir’s formula [41,42]. These results allow us to conclude that the Casimir forces between two plates are attractive in most of the cases reported so far.

### 2.2. Lens/Sphere–Plate and Sphere–Sphere Geometries with Different Material Combinations

A major difficulty in measuring Casimir force on the plate–plate structure is that the two plates cannot be kept perfectly parallel. One effective method is using a sphere or lens instead of one plate, and many studies have been conducted. Derjaguin et al. measured the attractive forces between fused quartz lens and plate, and the results agreed with Lifshitz’s theory, shown in Figure 3a [43,44]. Then, Rouweler et al. tested the same structure but the material of fused silica as Figure 3b shows [45,46]. In addition, the measurement between the plate and sphere with the coated chromium layer was completed, as can be seen in Figure 3c [47]. After 1997, more precise measurements of attractive Casimir forces between sphere and plate with coated metal layer have been performed. In Figure 3d–f, the structures investigated by Lamoreaux [48], Mohideen and Roy [49,50], Harris et al. [51], Decca et al. [52], and Canaguier-Durand et al. [53] are shown. The Casimir force between the sphere/lens and plate always seems to be attractive. However, Banishev et al. found that magnetic properties of magnetic metal influence the force magnitude and suggested that it was possible to obtain Casimir repulsion by using ferromagnetic dielectrics [54]. Munday et al. found when the sphere–plate structure was immersed in a specific liquid, the Casimir interaction was possibly changed from attractive to repulsive [55,56].

When researchers considered the sphere–sphere boundaries, Kenneth and Klich showed that the forces between two conducting hemispheres related by reflection are always attractive, independent of the exact form of the bodies or dielectric properties (Figure 3g) [57]. Considering the two concentric spheres, Özcan claimed the Casimir force between them was always attractive (Figure 3h) [58]. Recently, Garrett et al. indicated attractive Casimir forces between two goad-coated spheres in their measurement (Figure 3i) [59].

### 2.3. Cylinder–Cylinder/Plate/Sphere Geometries with Different Material Combinations

Different measurements of attractive forces between crossed cylinders of mica and silica sheet were completed by Tabor, Rouweler, Winterton et al. separately, and the schematic is shown in Figure 4a [3,45,60]. Ederth measured attractive forces between gold-coated crossed cylinders as Figure 4b shows [61]. Mazzitelli et al. computed Casimir interaction between two perfectly conducting, infinite, concentric cylinders as Figure 4c shows. They found that when the radii of two cylinders are close to each other, the attraction of the outer cylinder dominates, and the inner cylinder tends to expand. When the radius ratio is approximately 3, the inner cylinder tends to compress [62]. Considering the slightly eccentric cylinders, Dalvit et al. obtained similar results compared to concentric cylinders and the scaling of the Casimir force with the distance between the sphere–plate and the configuration of the parallel plates [63]. Another special situation is paired micromirrors in Figure 4d. Akhundzada et al. found that two neighbouring curled shutters attached to each other in some cases, probably because of the Casimir attractive force [64]. Then, Kästner, Elsaka et al. continued the studies of the improvement of the yield of paired shutters by optimising the fabrication process and varying the layer thickness [65,66].

Similar to the sphere–plate structure, much corresponding research exists for the cylinder–plate system, as shown in Figure 4e. Emig et al. assumed that the cylinder and plate were made of perfect metals and found attraction by calculation [67]. Brown-Hayes et al. implemented measurements of cylinder–plate geometry with gold metallic surfaces, and the results showed attractive force between objects [68]. Figure 4f indicates a cylinder–sphere structure; the attractive force between them was theoretically derived by Teo [69].

### 2.4. Special Geometries with Different Material Combinations

Since Casimir forces strongly depend on geometry, many further attempts were made to study the character of Casimir forces on more complex shapes. Calculation from Brevik et al. indicates that attractive forces between two walls of perfectly conductive and dielectric wedges with an opening angle as Figure 5a shows [70,71,72]. Then, based on the corrugated plates displayed in Figure 5b, Emig et al. found lateral Casimir forces and a crossover with the normal attractive Casimir forces [73,74]. Corresponding experiments followed afterwards. Chen et al. observed lateral forces between a corrugated plate and a corrugated sphere; the sketch can be seen in Figure 5c [75,76]. Based on two metallic squares sliding between two metallic walls demonstrated in Figure 5d, Rodriguez et al. demonstrated nonadditive and nonmonotonic changes in the forces [77]. Furthermore, their results showed that the transformation from attractive to repulsive force was concluded in the study on a glide-symmetric geometry, which consists of parallel plates with interleaved metal brackets (in Figure 5e) [78]. Levin et al. found repulsive forces in a new geometry consisting of an elongated metal particle centred above a metal plate with a hole, illustrated in Figure 5f [79].

In Figure 5g a liquid exists between the objects. In specific cases, a change in the sign of the Casimir force can occur. The interaction can be attractive, not existing (zero Z) or repulsive. Munday and Capasso measured the Casimir force between the gold sphere and plate separated by ethanol, which is approximately 80% smaller than the structure in a vacuum [55]. When the medium changed to bromobenzene, they observed the repulsive force between the gold sphere and plate [56]. McCauley et al. assumed a silica sphere sits atop a perfect metal plane with a spherical indentation, which was immersed in bromobenzene. The Casimir force is observed to change its sign as the displacement between the surfaces of the sphere and indentation changes [80]. After that, Rodriguez et al. calculated the Casimir force between a Teflon sphere and a silicon sphere located above the semi-infinite slabs, and all the structures were immersed in ethanol. They found the attraction, repulsion and equilibrium at different separations [81]. Figure 5h illustrates a gold-coated sphere and a silicon surface with trench arrays, and the Casimir force between them was measured by Chan et al. They found significant deviations between the experiment and theoretical calculation, which proved the strong dependence of Casimir force on the shape of interacting geometries [82]. Messina et al. derived the exact sphere-grating Casimir interaction energy and found that a metallic grating can be used to increase both the normal and the lateral Casimir interaction [83]. Figure 5i illustrates a recent study in which Tang et al. show that the Casimir forces changed non-monotonically when the displacement between two silicon structures with T-shaped protrusions decreased, manifesting as attraction, repulsion and no interaction [84]. Then, Wang et al. measured the Casimir forces between two nanoscale rectangular silicon gratings using similar methods to Tang. They concluded that as the distance decreased, a geometry dependence and a novel distance dependence of the Casimir forces appeared before and after gratings interpenetrated each other [85].

Concluding Section 2, materials and geometries play a crucial role in the strength of the Casimir forces and the interaction between the two objects: attractive, repulsive or no interaction.

## 3. Main Measurement Methods: Review of Casimir Metrology

**Figure 6:**
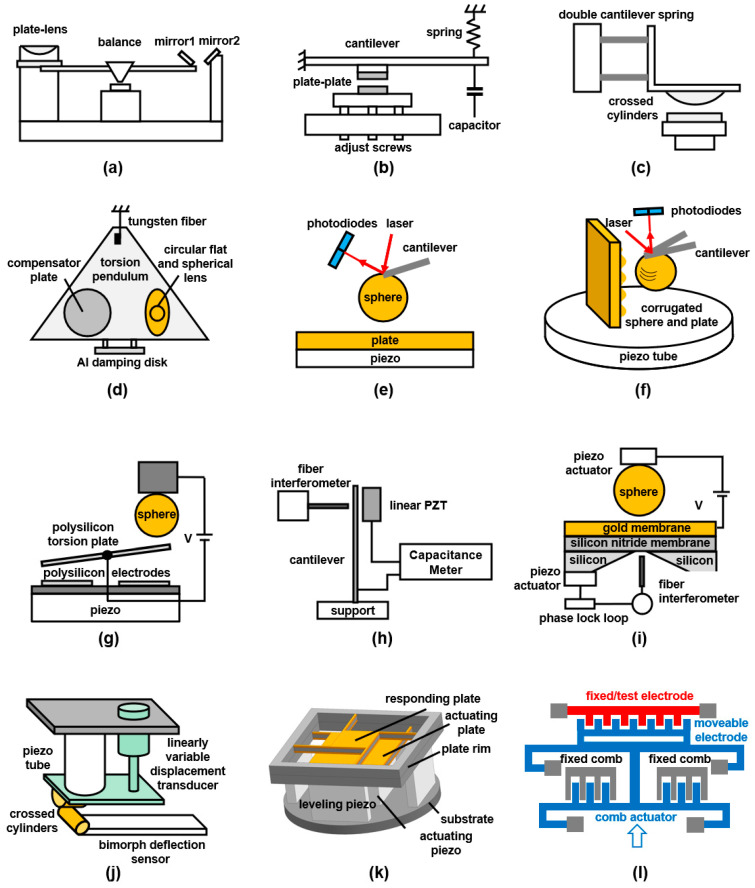
Main experiments of Casimir force. (**a**) Leverage system [43,44], (**b**) balanced levers system [33], (**c**) double cantilever spring system [3], (**d**) torsion pendulum system [48], (**e**) AFM system for plate–sphere [49,50,51,55,56,86,87], (**f**) AFM system for corrugated plate–sphere [75,76,88], (**g**) micromachined torsional devices [42,52,82,89], (**h**) fibre interferometer–cantilever system [41], (**i**) fibre interferometer–nanomembrane system [90], (**j**) piezoelectric tube–bimorph cantilever system [61], (**k**) vibrating plate system [91,92], (**l**) comb and amplifier system [84,85] (completely redrawn by the ideas of these references).

For the last approximately 80 years, many scientists and researchers have made significant contributions to the measurements of the Casimir force [25]. The first attempt at an experiment was the measurements of molecular attraction of solids by Derjaguin et al., employing a leverage system with shock absorbers, which is displayed in Figure 6a. They calculated the forces with the deflection of the leverage, which was detected by the reflection of two mirrors [43,44]. Then, Figure 6b indicates a cantilever system to measure the force between two flat plates by using adjust screws to decrease the distance. Sparnaay showed attractive forces by measuring the variation of capacitance but was unable to obtain accurate results because of some obstacles [33]. Tabor’s measurements found attractions between mica sheets cylinders, one of which was attached to the edge of a cantilever, converting from retarded to normal Van der Waals forces by the jump distance method [60]. Together with Tabor, Israelachvili measured the Van der Waals force at a wider range using a double cantilever spring system through a resonance method, as shown in Figure 6c. Their results displayed a gradual transition between normal and retarded forces as the separation increased [3].

In 1997, the interaction between a sphere and a plate with coated Cu and Au layers was detected by Lamoreaux, employing an electromechanical system based on a torsion pendulum, which can be seen in Figure 6d [48]. It was the first conclusive demonstration of Casimir forces. Then, by using an atomic force microscope (AFM) shown in Figure 6e Mohideen and Roy connected a metal sphere to the end of the cantilever and measured the deflection to calculate the force [49,50]. This method has been applied to more precise measurements of metal sphere–plate surfaces [51,86,87]. Munday and Capasso measured the Casimir force between two gold surfaces immersed in a fluid, and they experimentally proved that repulsive forces exist when the medium is not a vacuum [55,56]. Figure 6f illustrates the measurements performed by Chen, which were measurements of lateral forces between a corrugated plate and a sphere using AFM. The force showed periodicity corresponding to the corrugations [75,76]. Then, the lateral Casimir forces that arise between aligned sinusoidally corrugated surfaces of a sphere and a plate were measured by Chiu [88].

Another important measurement tool is a micromachined torsional device, as shown in Figure 6g. Chan et al. measured the separation between a polysilicon plate and a metallic sphere by the rotation angle of the plate and found a good agreement between experiments and theories. The results also showed that the quantum electrodynamical effects play an essential role in MEMS for nanoscale separations [89]. A few years later, they measured the interaction between a gold sphere and a silicon surface with nanoscale trench arrays in this way [82]. This method was also used by Decca et al. to measure the interaction between the sphere–plate with dissimilar metals as well as between two gold-coated plates [42,52]. Figure 6h illustrates a method to measure Casimir force between a silicon cantilever coated with chromium and a similar rigid surface by Bressi et al. The shifts of the cantilever frequency were detected when the rigid surface was approached [41]. Garcia-Sanchez et al. built a Si_3_N_4_ nanomembrane as a pressure sensor, which was under the gold plate (in Figure 6i). Using a fibre interferometer to measure the nanomembrane displacement, the Casimir force could be more precisely detected [90].

A system consisting of the piezo tube for reducing the distance, a linearly variable displacement transducer for corrections, crossed cylinders for actuators, and a bimorph cantilever for measuring deflection was presented by Erdth [61]. The structure was used for the measurement between crossed cylinders, and the schematic is shown in Figure 6j. As illustrated in Figure 6k, Nawazuddin et al. designed a vibrating plate–plate system to detect any asymmetry in the vibration modes of two plates to calculate the Casimir force between the parallel plates [91,92]. Figure 6l shows a system including fixed and movable electrodes, and the latter were actuated by movable combs. Tang et al. and Wang et al. measured the frequency response of the test electrode after amplification and then calculated the Casimir force between two surfaces with nanoscale protrusions [84,85]. Investigating the basic idea of Casimir and Lifshitz is based on parallel plates. Many experimental groups reported that it is extremely difficult to maintain sufficient parallelism between the plates. Therefore, using one plate and one sphere on a cantilever seems to be a good idea. However, this introduces additional degrees of freedom into the theoretical model calculations and increases the number of free parameters—not much, but noticeably.

## 4. Influence of Further Parameters on Casimir Forces

In Section 2, the influence of different geometries and materials has been reviewed, and in Section 3, different measurement methodologies for Casimir forces have been presented and compared. However, in addition to materials and geometries, many other parameters (factors) have a large influence on the Casimir forces. Section 4 considers the influence of temperature, conductivity and surface roughness [18,25]. The influence of those parameters has to be assumed to exist because the results from theoretical and experimental studies reveal disagreements. Theoretical model calculations can never be complete without generating huge numbers of free (unknown) parameters. Therefore, simulations have to neglect a lot of effects to keep the computation times in a reasonable frame. Adding an additional effect (neglected previously) into the considered model is often called “correction to the model or improvement of the model”. In the following, the potential influence of three additional critical effects are considered: (i) thermal issues, (ii) conductivity issues and (iii) surface roughness issues.

### 4.1. Influence of Thermal Effects

In the early stage of Casimir’s studies, Lifshitz studied the influence of temperature on molecular attractive forces and predicted that the effect is very tiny for small separations but substantial for large separations [8]. Then, Mehra gave more details that the thermal effect on the interaction between two plates was negligible for separations of 0.1–2 μm, but it became considerable for separations greater than 3 μm. Especially at 5 μm, the Casimir force was at least 50% higher than the one without corrections (model improvements by also including thermal effects) [93]. Sushkov et al. observed from experiments that the Casimir force, including temperature dependences, dominates at the separation between gold-coated plates over cavity thicknesses over 3 μm [94]. The Casimir effect between dielectrics with parallel surfaces for arbitrary temperature was reconsidered by Schwinger et al. [32], which confirmed Lifshitz’s results.

### 4.2. Influence of Conductivity Effects

Schwinger et al. observed small repulsions between the metal plates and speculated that it was due to the influence of a metal oxide layer with finite conductivity [32]. Lamoreaux calculated the Casimir force between imperfectly conducting plates by a simple plasma model but found a high deviation [95]. Lambrecht studied the influence of finite conductivity of metals, and the results showed that reduction in the Casimir force is different for flat plates of different materials [96]. Results from de Man et al. indicated that surfaces with conductive oxides had lower Casimir force than the noble metal [97]. From the measurement of Laurent et al., the Casimir force is known to be sensitive to the dielectric properties of the interacting surfaces [98].

### 4.3. Influence of Surface Roughness

It is well known that, in reality, it is impossible to have perfect surfaces, especially at the microscopic scale the surface irregularities are more pronounced, which is known as roughness. Van Blokland and Overbeek noticed the experimental resultant forces between two macroscopic objects of fused silica in a distance range of 20 to 260 nm were higher than the prediction of Lifshitz’s theory and pointed out that the roughness, which was in the range of 7 to 10 nm, must be taken into account [46]. Bree et al. derived an expression of interacting force with small surface irregularities, but the influence of different boundary conditions was quite large, ranging from 10 to 50% depending on whether we are in the retarded or nonretarded regime as well as the geometry itself [99]. Maradudin and Mazur calculated the interaction between rough parallel surfaces and concluded that higher roughness increases the forces in both cases if only one or the two surfaces are considered to be rough [100,101]. Moreover, the Casimir effect is more sensitive to roughness at short distances, causing forces to deviate from PFA calculations, as found by Genet et al. [102].

For more precise results of theoretical calculations and better agreement with the experiment, these three corrections should be taken into account. Bezerra et al. calculated the interaction of a lens placed above a flat plate of arbitrary size. It is shown that the corrections due to surface distortions (roughness) and finite conductivity have opposite signs and may compensate for each other [103]. Klimchitskaya et al. completed Casimir force measurement of sphere–plate geometry using AFM, and the result showed good agreement with calculation, including roughness and conductivity corrections [86]. Genet et al. gave numerical evaluations of the Casimir effect for plane metallic mirrors with temperature and conductivity effects treated simultaneously [104]. Then, they introduced a more general description with roughness sensitivity to achieve the desired level of accuracy in the theory–experiment comparison [102]. A precise measurement of the Casimir force between dissimilar metals showed very high agreement with the theoretical model that considered finite conductivity and roughness presented by Decca [52]. Chen et al. pointed out in their studies that the influence of roughness was more than over finite size and thermal effects [87]. Then, Mohideen and Roy calculated the Casimir effect again, considering all these three corrections, which were consistent with their experimental results [49,50].

Considering the research discussed in this section, a rough and simple summary can be comprised that, in general, both high roughness and high temperature increase the Casimir attraction (at a separation distance of about 0.5 μm the effects of temperature can be neglected [105]), while different conductivities of materials reduce the attractive force.

Overall, there is no doubt that surface roughness plays a significant role in the context of Casimir forces. This is usually considered by roughness corrections. These are typically based on a single parameter, which characterises the amplitudes of a surface topography obtained by atomic force microscopy [18,87]. However, the magnitude of rough surface amplitudes may differ from single nanometres [87] to values of the order of 30 nm rms amplitude for high-quality optically polished surfaces [18]. Another critical parameter not considered by the roughness correction is the averaged period or the correlation length of the surface topography, which needs to be either much larger or much smaller than the separation between the two objects affected by Casimir forces in order to allow geometrical averaging [18]. However, according to [87], typical grain sizes in a polycrystalline metal film are in the order of 70 to 120 nm and are thus in the same order as the separation, which ranges from 62 to 90 nm. Determining roughness effects, even under these conditions, requires solving the wave equation with rough boundaries, as mentioned in [18], as one of many options.

## 5. Influences of Casimir Forces on the Characteristics of MEMS/NEMS Devices

Typically, the orders of magnitude of Casimir force in micro-Newton level. At the micro- and nanoscale, the Casimir force has significant impacts on the performances of the devices, including many kinds of static as well as dynamic characteristics as follows [20].

### 5.1. Influences of Casimir Forces on Stiction and Adhesion

Stiction, which means adhesion due to the high friction created between two contacting solid surfaces, is one of the most important issues relevant to MEMS reliability [106]. In those locations where the distances are small, the increase in a Casimir force leads to stiction more easily [107]. One of the critical reasons for the increasing Casimir force is attributed to the greater surface roughness [108,109]. Membrane strips with a large aspect ratio (length/thickness) and low elastic modulus are also vulnerable to stiction [110]. During the crystallisation, the phase change materials could increase the Casimir force, and at the same time, the reduction of stiffness of the actuator can also lead to stiction [111]. Moreover, the effect of the Casimir forces on adhered cantilevers was considered by Svetovoy et al. [112]. It was concluded that Casimir forces only operate very close to the point of contact. However, these forces affect the shape of a portion of the structure, i.e., one-third of the length of the unadhered cantilever from this point.

### 5.2. Influences of Casimir Forces on Pull-in Voltages

Pull-in voltage is one of the most important nonlinear characteristics of MEMS devices with wide applications and limitations [113]. The pull-in voltages of micro- and nanodevices are typically low and inversely proportional to the square of the distance between the electrodes. The Casimir attraction between geometrical structures shortens the distance and increases the corresponding electrostatic force, leading to a reduction of the pull-in voltage. Highly nonlinear pull-in voltage variations considering Casimir effects have been demonstrated based on the theoretical derivations and experiments with cantilevers, fixed-fixed beams, electrostatic torsional actuators, micromembranes, oscillators and a variety of other MEMS/NEMS devices [114,115,116,117,118,119,120,121,122,123,124,125,126,127,128]. As the geometric parameters are greater or less than some particular values, the pull-in gap, as well as voltage with and without Casimir force, are obviously different [122,123]. Even in some cases, the pull-in phenomenon occurs with zero or very small driving energy input [116,128]. But at larger separations, the influence on the voltage becomes smaller [126]. Furthermore, the effect of the inertia of the device on the pull-in voltage increases significantly when Casimir forces are taken into account [124].

### 5.3. Influences of Casimir Forces on Vibrational Properties

It has been demonstrated that Casimir forces significantly impact the vibrational characteristics of MEMS/NEMS devices. Casimir forces reduce the stiffness of the MEMS structures (e.g., microcantilevers and circular micro-plates), which leads to a decrease in the resonant frequencies and an obvious increase in the resonance amplitudes [129,130]. According to studies on cantilevers, the influence on the frequency of the Casimir force also strongly relies on the geometry of the cantilever tip [131]. Chan et al. observed force-induced frequency shifts, hysteretic behaviour, and bi-stability in the frequency response of a micromachined torsional oscillator [132]. Additionally, combined with Casimir force, material gradient, size effect, geometric nonlinearity and axial residual stress significantly affect MEMS’s vibrational properties [133,134].

### 5.4. Influences of Casimir Forces on Chaotic Motion

The irregular motion of simple systems is called chaos, which means simple equations can have very complicated solutions [135]. At separations between plates of MEMS oscillators below 100 nm, it was demonstrated by Broer et al. that the nonlinearity of the Casimir force can cause chaotic motion, and the high roughness increases the possibility of this phenomenon [109]. Chaotic behaviour poses a significant risk of stalling and is more pronounced in conducting systems with increasing Casimir forces and torques [136]. When considering the oscillator with phase change materials, the crystalline phase transitions increased the Casimir force, leading to stronger chaotic behaviours [137]. In a vibrating noncontact rack and pinion, the lateral Casimir forces generated by the corrugated surface were able to reduce the chaos [138]. From another study on sphere–plate structure, higher lateral Casimir force caused by higher conductivity materials appeared to be a better choice to ensure stable operation against a chaotic movement [139].

In summary, considering the properties discussed above, the Casimir forces normally increase the possibilities of stiction and chaotic motion (except the lateral Casimir forces attenuate the chaotic movement), but decrease the pull-in voltages and the resonance frequencies.

## 6. Applications of Casimir Forces in MEMS/NEMS Field

A large number of publications describe the wide range of applications and potential future possibilities of the Casimir effect [140,141]. Since quantum effects play an important role in small separations between structures, they could provide new possibilities for novel actuation schemes for nanoelectromechanical systems based on the Casimir forces [89,117].

### 6.1. Highly Sensitive Sensors

Anharmonic Casimir oscillators (parallel plates) [142] and torsional resonators (sphere–plate geometry) [143] can serve as excellent platforms for designing highly sensitive sensors and detectors due to their extreme sensitivities and stabilities. Iannuzzi et al. designed a plate structure immersed in liquid and suspended on a surface due to the repulsive forces, which can be used to develop ultrasensitive force and torque sensors [144]. Furthermore, Miri and Golestanian proposed a nanoscale mechanical device consisting of two racks and a pinion driven by lateral Casimir forces, which could potentially serve as a mechanical sensor or amplifier [145].

An advanced application of sensors designed based on the Casimir effect is quantum measurements. One Casimir cavity consisting of a sphere–plate structure was successfully integrated into an MEMS system, and it was shown that it is possible to perform susceptible quantum measurements with off-the-shelf consumer MEMS sensors [146]. Based on a plate–sphere structure, Javor et al. designed an MEMS quantum-enhanced gradiometer, which paved the way for measuring extremely weak gradient magnetic fields [147]. Then, they demonstrated a new class of quantum sensors using an MEMS parametric amplifier and Casimir vacuum for the zeptometre positional sensing [148].

### 6.2. Non-Contact Actuators

The Casimir force can also be exploited as a driving force for many special structures. Ashourvan et al. developed a noncontact rack–pinion structure powered by the lateral Casimir force [149]. Using the lateral or normal component of the Casimir force through suitably constrained moving parts and designed surfaces, Carter et al. demonstrated a non-contact actuation of comb structures in the wafer plane [150]. Zhao et al. found a stable Casimir equilibrium between a gold nanoplate and a Teflon-coated gold surface, which can be used as a platform for a variety of applications such as contact-free nanomachines and nanoscale manipulations [151].

### 6.3. Stiction-Related Applications

The stiction induced by the Casimir forces is omnipresent and, in addition to causing the device’s failure, adhesive forces can be controlled and implemented under specific conditions [152]. A well-known example of the application is a gecko attached to a smooth surface. This is because a gecko’s foot has nearly five hundred thousand keratinous hairs or bristles, which provide strong adhesion [153,154]. Adhesion caused by the Casimir forces is available to be applied in the fields of particle control and removal [155,156].

### 6.4. Heat Transfer Devices and Actuators

The ability to control thermal flow using quantum vacuum paves the way for the study of quantum thermodynamics, implementing quantum thermal machines and the exploitation of quantum vacuum in energy transport at the nanoscale [157]. Tercas et al. proposed a quantum heat machine consisting of two nanomechanical resonators powered by the Casimir interaction [158]. Fong et al. experimentally discovered an unknown mechanism of heat transfer driven by quantum fluctuations. Controlling strong Casimir phonon coupling provides a versatile platform for realising coherent phonon processes (e.g., phonon state transfer and entanglement) using a quantum vacuum [159].

### 6.5. Optical Applications

Casimir force also has great potential for optical applications. The measured Casimir torque between two optically anisotropic materials suggested the potential for the torque to be used as a micro- or nanoscale actuation mechanism [160]. Considering the optomechanical system with tuneable Casimir forces, Liu et al. found that the optical output rate can be significantly altered, opening up the possibilities of designing exotic optical nano-devices by harnessing the power of a vacuum [161]. In addition, switching Casimir forces with phase change materials in a few nanoseconds is very useful in electronic and optical memory applications [162].

### 6.6. Harvesting Devices

The Casimir machine, consisting of a non-contact rack and pinion, acts as a successful kinetic energy harvester, which was studied by Miri and Etesami [163]. Román-Ancheyta et al. studied the dynamical Casimir effect in stochastic systems that can harvest photons through the noise [164].

### 6.7. Applications in Quantum Computation and Communication

The connection between the dynamical Casimir effect and the performance of quantum information protocols was illustrated by Benenti et al. Since the ultra-strong regime has already been investigated in circuit quantum electrodynamics experiments, it can be predicted that the dynamical Casimir effect has great advantages in applications of quantum computation and communication [165].

### 6.8. The Role of Machine Learning

Typically, scientific experiments are designed using a step-by-step linear approach that includes planning, execution, data processing, evaluation, and interpretation. A key challenge in taking experimentation to the next level is to immediately use the findings from an evaluation/interpretation to plan the next experiment by creating a feedback loop between the last and first steps. Online data analysis is essential for this, which is why artificial intelligence, especially machine learning, should be used. In addition to near-real-time evaluation, this approach has the potential to make experiments smarter, faster and more efficient and to make them controllable [166]. For experiments with MEMS/NEMS in particular, this means being able to carry out more targeted experiments to observe expected effects (e.g., Casimir effects). In addition to experimentation, simulations can be used to gain new insights and thus open up potential fields of application for these techniques. Instead of using complex FEM simulations, for example, fast simulations with surrogate models based on machine learning can be used.

## 7. Self-Assembling Structures

Casimir forces provide the possibility of combining two or more structures together and achieving special functions. The new structures are usually named self-assembling structures because there is no additional input except the Casimir force. Akhundzada et al. developed self-assembled 3D MEMS arrays with paired cylindrical metal layers, which have interesting potential applications in microfluidic diagnostics and medical drug delivery [64,65]. Munkhbat et al. introduced an approach to micron-scale self-assembly based on the joint action of attractive Casimir and repulsive electrostatic forces arising between charged metal nanoflakes in an aqueous solution. This system forms a self-assembled optical Fabry–Pérot microcavity with a tuneable equilibrium configuration that can be used as a sensitive and tuneable platform for a variety of applications, including optomechanics, nanomechanics, and cavity-induced polaritonic chemistry [167]. Other interesting research fields about self-assembling are micro-origami and micro-kirigami, which are inspired by the traditional Japanese art of paper-folding and normally structured by surface tension. They provided additional degrees of freedom in creating unprecedented 3D geometries, beyond the limitations of the conventional method [168]. Høye and Brevik revealed that the work completed by the Casimir force when the separation between the plates changes precisely reflects the surface tension of the plates [169], which means that the application of the Casimir force in Origami and Kirigami is possible.

Three-dimensional self-assembly at the micro- and nanoscales requires the spontaneous movement of system components to be organised to form a specific geometry without external interference. Depending on the medium being worked on, 3D self-assembly can be achieved through various techniques and within different systems. Those techniques may include intermolecular forces, templating techniques, inducing intrinsic stress or applying external stimuli. Here we shortly provide several examples of such a process. DNA Origami is a process where a single strand of DNA molecule can be folded on itself to form different 3D geometries such as cubes or spheres, and that is through the design of the complementary base pairing [170]. Block copolymer self-assembly is a technique involving two or more chemically distinct polymer chains that are covalently linked together; they can self-assemble into various geometries, such as spheres or cylinders, driven by the thermodynamics forces and by controlling the composition and the molecular weight of the polymers as well as the processing conditions [171]. Colloidal self-assembly involves colloidal particles that are suspended in a solvent that can self-assemble into ordered structures driven by interparticle interactions such as electrostatic, Van der Waals, or steric forces. By manipulating the particle size, surface chemistry and shape, the range of 3D geometries can be expanded. In template-assisted self-assembly, templates can be used to guide the self-assembly of nanostructures into specific geometries. These templates can be physical, e.g., nanopatterned surfaces, or chemical, e.g., molecular templates. By controlling these templates and their interaction with the self-assembly components, 3D geometries can be achieved [172]. In capillary self-assembly, capillary forces can drive the self-assembly of micro and nanostructures into specific geometries based on the components’ shape and surface. In external field-driven self-assembly, external fields can be magnetic, electric fields, or even light to manipulate and control the self-assembly of micro and nanostructures into desired geometries [173]. Those methods show diverse approaches used to achieve 3D self-assembly of different geometries at the micro and nanoscale, with vast applications in optoelectronics, photonics, drug delivery and tissue engineering [174].

### Three-Dimensional Self-Assembly into Yin–Yang Structures

**Figure 7:**
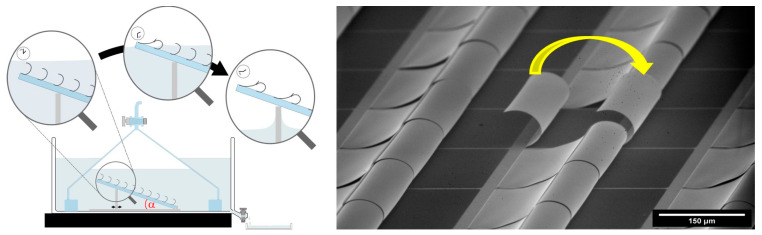
Shows the drying process in which the microshutters come together to form the Yin–Yang structure on the left-hand side, and on the right-hand side, an SEM micrograph of the resultant paired shutters. Modified from [64,65].

The previous section discussed the fact that 3D self-assembly is quite an interesting application and that different techniques can be used there, one of which is the Casimir forces. In this section, we show and discuss a special structure, namely Yin–Yang-paired shutters, with an overview of their fabrication process as an MEMS process with involved parameters and the potential role of Casimir forces in obtaining such promising structures, revealing a connection interplay between MEM systems, Casimir forces, and 3D self-assembly. The main principle and process were first stated in [64], which shows a detailed fabrication process, Casimir force simulation, and calculation. Here, we revealed the role of the final step of the fabrication process, drying, which helps bring two microshutter blades in close proximity to each other, allowing the Casimir forces to come into the picture and allowing the two shutters to self-assemble into the Yin–Yang structure. The drying set-up can be seen in Figure 7.

**Figure 8:**
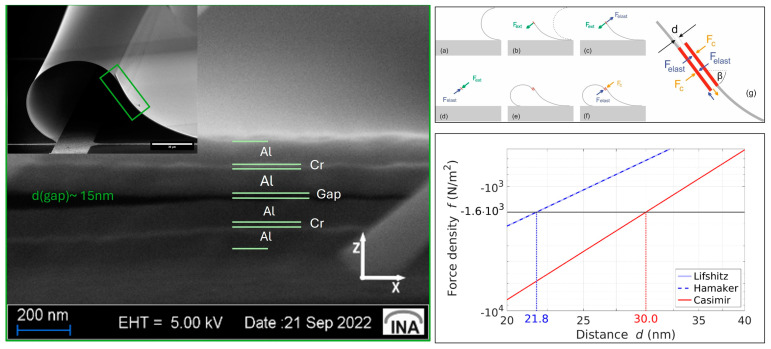
**Left**: Focused ion beam micrograph of the area where two microshutters come close together with a gap around 15 nm. **Top right**: Comsol simulation of the steps to estimate the distance d. (**a**) Un-actuated shutter, (**b**) un-actuated shutter (dotted) and shutter (full line) actuated via an external force F_ext_ acting on the area A (its cross-section highlighted as a red stripe), (**c**) elastic force F_elast_ and counteracting external force F_ext_ on area A, (**d**) the identical force equilibrium with the same but shifted forces, (**e**) both shutters in grey overlapping within A (red), (**f**) force equilibrium for the right shutter: restoring elastic force Felast and counteracting Casimir force F_C_, acting on the right area A (red), (**g**) force equilibrium also involving forces acting on the left shutter and formation of a plate capacitor arrangement (red) with known area A and distance d to be determined. **Bottom right**: Model calculations of the obtained Casimir force densities depending on the distance between the shutter blades d for (1) the Casimir approach (red line), (2) the Hamaker approach (dashed blue line) and (3) the exact model (solid light blue line), respectively. Modified from [64].

Focused ion beam (FIB) etching and scanning electron microscopy (SEM) reveal an average gap of approximately 10 nm between overlapping shutter blades. Quantitative analysis using COMSOL Multiphysics simulations indicates that a Casimir force of 9 × 10^−6^ N is required to counteract the restoring elastic forces for an area of 5600 µm^2^, implying a sufficient gap distance of less than 30 nm, see Figure 8. Further calculations adjusting for realistic material properties and non-ideal conditions confirm that the actual gap distance of 10 nm produces a much larger Casimir force, indicating the shutters are pressed together more than ten times stronger than the minimum required force. This force density significantly increases as the gap distance decreases, affirming the tight attachment of the shutters through Casimir forces, which are quantitatively consistent with the observed stability and configuration of the paired shutters.

To understand the relevance of the various forces that may exist in the micro/nanoscale, in particular in the context of MEMS/NEMS, we analyse and compare gravitational, electrostatic, capillary, and Casimir forces. This understanding is pivotal for applications such as 3D self-assembly, where they play a crucial role in manipulating micro and nanostructures [175]. For this, we use the paired MEMS shutter geometry as the basis for our force calculations.

System configuration as an assumption for our model:

- Dimensions A = 400 µm × 14 µm = 5600 µm^2^.
- Mass per unit area: $\sigma_{m}$ = 0.62 g/m^2^ considering the Al-Cr-Al metal stack.
- potential difference: U=1 mV (due to charge fluctuations based on deviations in the working function of the facing aluminium surfaces).
- Contact angle: θ ≈ 20° (for isopropanol on hydrophilic aluminium).
- Surface tension: $\gamma_{l}$ = 17 mN/m (for isopropanol at 68.5 °C) [176].
- Hamaker constant: $H=4.554\times 10^{-19}$ (for aluminium) [64].

The force per unit area is calculated in dependence on the distance d, assuming d≪A.

1. Gravitational force per unit area:

$$f_{grv}=\pi G\sigma_{m}\frac{1}{d^{2}}$$

where $G=6.67430\times e^{-11}$ m^3^/(kg s) is the gravitational constant.

The gravitational force is typically very weak at micro- and nanoscales due to its small mass and quadratic dependence on the distance *d*. It is often negligible compared to other forces.

2. Electrostatic force per unit area:

$$f_{el}=\frac{1}{2}\varepsilon_{0}U^{2}\frac{1}{d^{2}}$$

where *ϵ*_0_ = 8.854 × 10^−12^  F/m is the vacuum permittivity, and *U* is the potential difference.

The electrostatic force, while stronger than the gravitational force, is influenced by the potential difference. It scales inversely with the square of the separation distance, making it significant at very small distances.

3. Capillary force per unit area:

$$f_{cap}=2\gamma_{l}\cos\theta \frac{1}{d}$$

where θ ≈ 20 °C and $\gamma_{l}=17$ mN/m [176].

Capillary forces arise due to surface tension effects and become prominent when there is a liquid interface [177]. They scale inversely with the distance *d*, and their magnitude can dominate in micro-systems where surface phenomena are crucial.

4. Casimir force per unit area in retarded limit (original formula of H. B. G. Casimir):

$$f_{Cas}=\frac{\pi^{2}\hbar c}{240}\frac{1}{d^{4}}$$

where ℏ is the reduced Planck constant, and c the speed of light.

5. Casimir force per unit area in non-retarded limit (based on VdW interaction):

$$f_{vdW}=\frac{H}{6\pi }\frac{1}{d^{3}}$$

where H is the Hamaker constant evaluated for aluminium [64].

The Casimir force, particularly in the non-retarded limit, becomes extremely significant at nanoscales due to its strong dependence on distance. This force is a key player in MEMS and NEMS applications where quantum effects are non-negligible.

**Figure 9:**
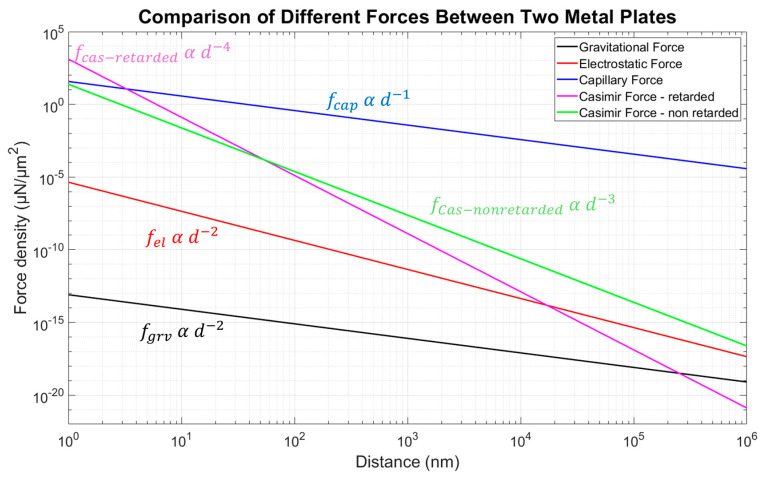
Graph showing comparison between different forces including gravitational, electrostatic, capillary and Casimir (retarded and non retarded) forces based on the introduced model. On the horizontal axis is the separation distance between the metal plates and on the vertical axis is the force density. The black line represents the gravitational forces, the red line represents the electrostatic forces, the blue line represents the capillary forces, the green line represents Casimir forces in the retarded regime and the magenta is the Casimir forces in the nonretarded regime.

In MEMS and NEMS, the Casimir and capillary forces (and electrostatic force for elevated potential fluctuations) are of tremendous importance due to their influence on device operation and stability. The Casimir force, in particular, plays a crucial role in nanoscale systems, affecting component adhesion and friction. It is also pivotal in the self-assembly processes of complex structures like the Yin–Yang geometry, where precise control of micro- and nanoforces is required to achieve desired configurations. Overall, while gravitational forces are negligible in microscale systems, electrostatic, capillary and Casimir forces play dominant roles, as seen in Figure 9. Their understanding and manipulation are essential for the design and functioning of MEMS/NEMS devices and for the 3D self-assembly of intricate structures.

Capillary forces play a crucial role in initiating the movement of paired MEMS shutters towards each other [65]. These forces act as a significant driving mechanism that induces the pairing process. Without the influence of capillary forces, the initial movement and subsequent pairing of the shutters would not occur. During the drying process, which is performed at elevated temperatures, the isopropanol liquid completely evaporates. Consequently, the capillary forces cease to act as a connecting medium between the paired shutters. Despite this, the configuration remains stable even at temperatures significantly exceeding the boiling point of isopropanol, as demonstrated by tests conducted on a hotplate at temperatures exceeding 150 °C.

**Figure 10:**
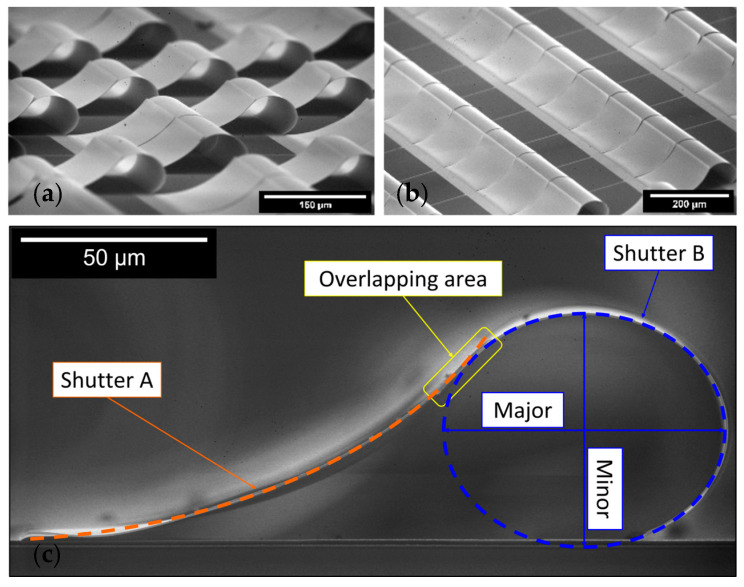
Paired shutter arrangement as checkerboard (**a**) and tubes (**b**). The pairing and the overlapping area between the shutter blades, A and B, and the fitted orange and blue ellipses to identify both of the shutter blades. The extracted fit parameters are the major and minor axes (**c**).

In a follow-up study [64], we focused on the investigation of the drying step assembling the two paired shutters. More specifically, the dependence of the yielded paired shutters on the tilt angle of the sample during the drying was studied over a range between 0° and 90°. In conclusion, it was shown that the pairing yield is dependent on the tilt angle: the higher the angle, the higher the yielding peaking at an angle of 40° and decreasing again for higher angles. Further study explored the geometry changes when varying the intrinsic stress of the shutters by varying the thickness of the lower Al layer, focusing on unpaired and paired geometries [66]. The hybrid stack, comprising aluminium and chromium layers, induces curling in freestanding shutters whose curvature decreases nonlinearly with increasing aluminium layer thickness (d_Al2_). Paired shutters form Yin–Yang shapes under Casimir forces, with geometries characterised by varying eccentricity (0.36 < ε < 0.67) and overlapping lengths (l_o_ ranging 8–31 µm) as shown in Figure 10. Three pairing scenarios are identified based on SEM and CLSM imaging, revealing nonlinear dynamics in the assembly process. The fabrication process involves layer deposition and sacrificial layer removal, enabling controlled assembly [42].

In our latest study [178], we present employing zirconium (Zr) as the building material of the paired shutters instead of (Al). Fabrication involves lithography, deposition, lift-off, and drying processes adapted to address challenges such as poor adhesion and film wrinkling specific to Zr. Optimal adhesion was achieved through oxygen plasma treatment and lithography adjustments, while film stability was enhanced by controlling deposition parameters. Challenges with high intrinsic stress causing excessive rolling of microshutters were mitigated by adjusting layer thicknesses and deposition conditions, aiming to achieve a desired radius of curvature for effective pairing. Overall, this proves that the pairing of the shutters is not dependent on the previously established building material of the system. For the scope of this paper, we focus on a single step, which is the lift-off and exploring the influence. The lift-off process is a patterning technique used to create intricate structures on substrates with high precision. It involves the deposition of a sacrificial layer, patterning this layer to define the structure features, and finally, removal of the sacrificial layer, leaving behind the patterned features. This technique provides several advantages, including high resolution, compatibility with various materials and the ability to create complex geometries. The process usually starts with preparing the substrate, which is generally made of silicon, any other semiconductor materials or glass in our case. A sacrificial material, typically a photoresist material, is deposited onto the substrate utilising spin-coating. Here, the sacrificial layer serves as a temporary support for the subsequent deposition of the system’s functional materials. Next and in a process known as photolithography, a photomask containing the desired pattern is placed over the sacrificial layer; the photomask, which is typically made of a material of chrome on glass, has the pattern with transparent areas, allowing light to pass through, and dark ones blocking the light. The substrate with the photomask on top of it is exposed to UV light at a certain exposure dose, which transfers the pattern from the photomask into the sacrificial layer. After that, the system’s materials, which can be metals, semiconductors or insulators, are deposited on the sacrificial layer. The deposition techniques include evaporation, chemical vapour deposition, plasma-enhanced chemical vapour deposition, etc. Once the material is deposited, the lift-off process can be initiated to remove the sacrificial layer. The sacrificial layer can be removed using a suitable solvent that selectively dissolves it without affecting the system material. This process allows the material to lift off from the substrate, leading to obtaining the desired structure. The choice of the suitable solvent is based on the type of the used material as a sacrificial layer, i.e., the photoresist material, either positive or negative photoresists, as well as the deposition technique used to deposit the system’s material, either sputtering or evaporation.

Initially, the fabrication process for obtaining the paired microshutters utilised 1-Methyl-2-pyrrolidon “NMP” as a solvent for lift-off at 80 °C hotplate temperature for 18 hrs. Due to NMP’s toxicity and unavailability, the lift-off solvent has to be changed. An alternative was dimethyl sulfoxide “DMSO”. It is stated that the lift-off solvents, in general, should not affect the fabricated device or the substrate during the process of dissolving the sacrificial layer; nevertheless, the process may need adjustment when switching from one solvent to another. The solvent was initially used at 80 °C, as recommended by the manufacturer; however, the paired microshutters were not obtainable under those conditions. It has been observed in a previous publication [64] that certain circumstances have to be there to assemble the two microshutters into paired ones, among which is the Radius of Curvature (RoC), which has to fall into a certain range between 30 and 60 µm. The resultant microshutters were freestanding, and their RoC was outside the required range. Hence, the process had to be modified by increasing the lift-off temperature to 90 °C instead of 80 °C, yielding the desired outcome. It has been determined that when using lift-off solvents besides DMSO and NMP, the pairing phenomenon is solely dependent on the operating temperature specific to each solvent rather than the solvent itself. Metal creep [179] is defined as a gradual deformation of the metal over time when it is subject to a constant load or stress, typically at a high temperature. When considering the lift-off process parameters temperature and timewise, it can be concluded that the process introduced a slight deformation pronounced as the change in the RoC when using different temperatures.

## 8. Summary and Conclusions

In essence, the Casimir effect represents a dynamic journey of scientific exploration, connecting theoretical insights with experimental realities to unravel a complex phenomenon. From Casimir’s initial prediction of an attractive force between conducting plates to Lifshitz’s broader interpretation involving dielectric properties, our understanding of Casimir forces has significantly evolved. Subsequent research, including contributions from Spanaary, Boyer, Derjaguin, and others, has not only confirmed the expected attractive interactions but also revealed intriguing instances of repulsion, highlighting the intricacy of these forces. The advancement of sophisticated measurement techniques, ranging from lever systems to atomic force microscopy, has empowered researchers to delve deeper into Casimir interactions, investigating diverse geometries and materials with unprecedented precision. However, this progress has also revealed the intricate dependencies on parameters such as temperature, conductivity, and surface roughness. Addressing these factors is crucial for precise theoretical predictions and experimental validation, guiding us towards a comprehensive understanding of Casimir effects. In MEMS/NEMS devices, Casimir forces have a significant impact, exacerbating challenges like stiction while also presenting opportunities for innovative applications. Despite their potential drawbacks, Casimir forces form the basis for highly sensitive sensors, non-contact actuators, and advanced fabrication techniques. Furthermore, the integration of machine learning methodologies holds the promise of enhancing research efficiency and opening up new avenues for exploration. Looking ahead, the Casimir effect continues to inspire curiosity and drive technological innovation. By navigating the complexities of Casimir interactions and harnessing their potential, researchers are poised to unlock new frontiers in science and engineering, paving the way for transformative advancements in diverse fields.

This review overlooks a wide variety of experimental and theoretical studies and focuses on four main aspects: the combinations of different materials, the considered geometries, the applied experimental measurement methodologies, and a survey of our novel self-assembly method. The disagreement between experimental data and the results of theoretical model calculations is often not satisfying. Although theoretical groups added several corrections (e.g., thermal effects) and could improve the agreement considerably, we state that non-ideal conditions in the experiments are also a major source of disagreements. Due to our own experiences with simulations, we state that mechanical calculations are, in most cases, operating with bulk (3D) material parameters. However, 2D values of permittivity, permeability, mass density and Young’s modulus are still not available with sufficient accuracy. That means that the parameters of ultra-thin layers show a significant dependence on layer thickness. In addition, quantum effects will also be game changers. This reminds us of the old debate in the early nineteenth about the use of 3D and 2D effective masses in strained semiconductor quantum wells, as well as the debate about the band offsets (not available precisely enough for different semiconductor material combinations).

Overall, it is highly interesting to see that by taking completely different theoretical models, the results can be similar or even identical. The latter concerns the power law of the gap distance and the Casimir and Van der Waals theoretical model calculations, which is one of the largest differences in defining the models. That means that the Casimir effect is of universal nature. It also shows that the Casimir forces and the Van der Waals forces are finally the same phenomenon, unified under the so-called dispersion forces [6]. Considering the quantum–electrodynamic model and the macroscopic permittivities of all involved media and the zero point energy of electromagnetic modes is the first model and one possible way to approach it. A second and completely different approach is to consider the microscopic dipole–dipole interactions between blades, which seem to be a completely different way; however, this also leads to power laws in the dependence of gap distance d. A third and astonishing way is the relativistic consideration of Van der Waals forces leading to the same result as Casimir found originally.

## Data Availability

Not applicable.
